# Bacterial oxygen production in the dark

**DOI:** 10.3389/fmicb.2012.00273

**Published:** 2012-08-07

**Authors:** Katharina F. Ettwig, Daan R. Speth, Joachim Reimann, Ming L. Wu, Mike S. M. Jetten, Jan T. Keltjens

**Affiliations:** Department of Microbiology, Institute for Water and Wetland Research, Radboud University Nijmegen,Nijmegen, Netherlands

**Keywords:** oxygen production, nitric oxide, nitric oxide reductase, chlorate reduction, chlorite dismutase, Cld, “*Candidatus* Methylomirabilis oxyfera”, strain HdN1

## Abstract

Nitric oxide (NO) and nitrous oxide (N_2_O) are among nature’s most powerful electron acceptors. In recent years it became clear that microorganisms can take advantage of the oxidizing power of these compounds to degrade aliphatic and aromatic hydrocarbons. For two unrelated bacterial species, the “NC10” phylum bacterium “*Candidatus* Methylomirabilis oxyfera” and the γ-proteobacterial strain HdN1 it has been suggested that under anoxic conditions with nitrate and/or nitrite, monooxygenases are used for methane and hexadecane oxidation, respectively. No degradation was observed with nitrous oxide only. Similarly, “aerobic” pathways for hydrocarbon degradation are employed by (per)chlorate-reducing bacteria, which are known to produce oxygen from chlorite $\left(ClO_{2}^{-}\right)$. In the anaerobic methanotroph *M. oxyfera*, which lacks identifiable enzymes for nitrogen formation, substrate activation in the presence of nitrite was directly associated with both oxygen and nitrogen formation. These findings strongly argue for the role of NO, or an oxygen species derived from it, in the activation reaction of methane. Although oxygen generation elegantly explains the utilization of “aerobic” pathways under anoxic conditions, the underlying mechanism is still elusive. In this perspective, we review the current knowledge about intra-aerobic pathways, their potential presence in other organisms, and identify candidate enzymes related to quinol-dependent NO reductases (qNORs) that might be involved in the formation of oxygen.

## INTRODUCTION

In dim anoxic waters of stratified lakes where oxygen-respiring organisms normally cannot survive, a tiny aerobic eukaryote nevertheless makes a living. This heterotrophic ciliate, *Histiobalantium natans*, can survive without external oxygen because it sequesters chloroplasts from ingested euglinoid flagellates (*Phacus suecicus*). The chloroplasts, kept active in the ciliate and surrounded by the mitochondria, photosynthesize and produce oxygen that allows the host to thrive in deep waters of stratified lakes, where it avoids metazoan predation and competition with other aerobic ciliates (Esteban et al., 2009). This is just one example of nature’s many twists that allow organisms to take a specific niche: If an essential compound is not available, make it yourself by inventing a variation on a general theme.

For a long time, photosynthesis was the only biological process known to produce oxygen. Cyanobacteria, green plants, and algae use light energy to split water$\left({E_{0}}^{\prime }=+0.82\,V\right)$ via photosystem II. The electrons obtained serve NADPH and ATP generation for carbon dioxide fixation; oxygen is a mere by-product of this metabolism. This pathway evolved at least 2.7 billion years ago (Canfield, 2005), and, after the vast pools of reduced compounds on early earth were exhausted, oxygen started to accumulate in the atmosphere around 2.45 billion years ago (Holland, 2006). As a consequence, organisms evolved numerous mechanisms to cope with and/or exploit its strong oxidative properties. To prevent oxidative damage by reactive oxygen species (ROS) like superoxide $\left(O_{2}^{-}\right)$, hydrogen peroxide (H_2_O_2_), or the most damaging of all, the hydroxyl radical (OH·), detoxification systems, which often result in the regeneration of oxygen (e.g., by catalase or superoxide dismutase) evolved. These reactions have been studied and reviewed in detail elsewhere (Apel and Hirt, 2004; Murphy, 2009) and are beyond the scope of this article.

On the other hand, a large number of extant organisms are completely dependent on oxygen as the terminal electron acceptor in aerobic respiration. In addition, oxygen is the substrate in an enormous variety of monooxygenase and dioxygenase reactions where one or two oxygen atoms, respectively, are incorporated into the substrate, either for degradation of compounds like aromatic and aliphatic hydrocarbons or biosynthesis of secondary metabolites.

*De novo* oxygen production can be driven by either light or chemical energy. The second, “dark” way takes advantage of oxidants with a more positive redox potential than the O_2_/H_2_O couple. Only a few redox couples are biologically relevant in this respect: hypochlorite(ClO^-^)/Cl^-^;${E_{0}}^{\prime }=+1.31\,V$, chlorite $(ClO_{2}^{-})/ClO^{-}\,({E_{0}}^{\prime }=+1.28\,V),\,ClO_{2}^{-}/Cl^{-}\,({E_{0}}^{\prime }=+1.08\,V),$ nitrous oxide $(N_{2}O)/N_{2}({E_{0}}^{\prime }=+1.36V),\,$ nitric oxide $(NO)/N_{2}O({E_{0}}^{\prime }=+1.18V),\,$ and $NO/N_{2}({E_{0}}^{\prime }=+1.27V).$ Most of these compounds are intermediates in the respiration of (per)chlorate and nitrate/nitrite, respectively. In this perspective, we review what is known and still to be learned about oxygenic pathways from chloro-oxo species and nitrogen oxides, with a focus on a hypothetical enzymatic mechanism for the hitherto elusive nitrite-driven oxygen production.

## OXYGEN PRODUCTION IN CHLORATE-REDUCING BACTERIA

The first group of oxygenic chemotrophs identified were perchlorate and chlorate respiring bacteria (Rikken et al., 1996; van Ginkel et al., 1996). These organisms reduce perchlorate $\left(ClO_{4}^{-}\right)$ and/or chlorate $\left(ClO_{3}^{-}\right)$ to chlorite$\left(ClO_{2}^{-}\right)$ . Rather than being further reduced to hypochlorite (ClO^-^), chlorite is converted into chloride (Cl^-^) and O_2_. Perchlorate occurs naturally, but rarely in the environment, with significant concentrations only found in the Chilean salpeter deposits (Beckurts, 1886; Ericksen, 1983). In past decades, anthropogenic contamination from either the use of Chile salpeters as fertilizers, or from chemical waste (e.g., solid rocket fuel spills and explosives) has been a concern and incentive for research on microbial (per)chlorate reduction (Motzer, 2001; Xu et al., 2003). An initial surprise was the wide-spread occurrence of (per)chlorate reduction among microorganisms and in different ecosystems, much broader than could be expected from the known natural sources and the short timeframe of anthropogenic contamination (Coates et al., 1999). It now has become clear that perchlorate is continuously generated in trace amounts in the atmosphere. Accumulation to measurable amounts, however, only occurs where deposition is high, but leaching and microbial reduction is low: in an extremely arid climate (Rajagopalan et al., 2006; Kounaves et al., 2010).

(Per)chlorate respiration in principal only requires two enzymes. At first, (per)chlorate reductase, a molybdopterin-containing respiratory reductase resembling nitrate reductase, catalyzes the reduction of perchlorate to chlorate and of chlorate to chlorite. Then, chlorite is converted in a single exergonic reaction into chloride and oxygen (Eq. 1; van Ginkel et al., 1996). This is a net disproportionation or dismutase reaction in which the chlorine atom becomes reduced and oxygen oxidized.

$$\begin{array}{cc} ClO_{2}^{-}\to Cl^{-}+O_{2}\left(\Delta G^{0^{\prime }}=-100\,kJ\,mol^{-1}O_{2}\right) & \left(1\right) \end{array}$$

The reaction is catalyzed at a high rate and with extraordinary specificity by chlorite dismutases (Cld, EC 1.13.11.49), members of the recently defined CDE superfamily of heme enzymes (Goblirsch et al., 2011). This homohexameric or homopentameric heme *b* enzyme was first purified in the nineties from the β-proteobacterium *Azospira oryzae *(then called strain GR-1, van Ginkel et al., 1996). The enzyme is now well characterized by the resolution of the atomic structures from several species (de Geus et al., 2009; Mehboob et al., 2009b; Goblirsch et al., 2010; Kostan et al., 2010; Mlynek et al., 2011). Detailed kinetic analysis established that oxygen is not derived from water, but from chlorite itself (Lee et al., 2008; Streit and DuBois, 2008). Catalysis proceeds via an oxoferryl species and a ClO^-^ anion, indicating that, after initial binding to the catalytic heme *b*, chlorite is first cleaved, after which both oxygen atoms recombine, yielding chloride and oxygen. Most chlorate-reducing organisms found thus far are facultative aerobes that, in absence of extracellular oxygen use the chlorite-derived oxygen for aerobic respiration, analogous to the use of chloroplasts by *H. natans* in the introduction.

What else to do with oxygen produced by chorite dismutation? Surprisingly, functional chlorite dismutases have also been found in Bacteria and Archaea that cannot grow with chlorate as electron acceptor, e.g., in the nitrite-oxidizing genera *Nitrospira* (Maixner et al., 2008) and *Nitrobacter* (Mlynek et al., 2011). In the latter species, Cld is significantly smaller and present as a homodimer. The role of Cld in these organisms, however, is unclear. They may possibly confer insensitivity to chlorite, coupled with the advantage of producing oxygen for nitrite oxidation in microoxic niches. In the archaeon *Haloferax volcanii*, chlorite dismutase is hypothesized to produce oxygen for a monooxygenase encoded in the same operon, that is involved in the biosynthesis of an antibiotic (Bab-Dinitz et al., 2006). In *Pseudomonas* chloritidismutans, oxygen likely does not only act as an electron acceptor in respiration, but is also used for alkane activation by a monooxygenase-mediated reaction (Mehboob et al., 2009a). *P. chloritidismutans* is capable of respiring carbon substrates like fatty acids or alcohols with oxygen, chlorate, and nitrate, but growth on alkanes is not observed with nitrate as electron acceptor. This suggests that oxygen, provided externally or from chlorate reduction, is required for the initial activation of the alkane, a hydroxylation to the corresponding alcohol (Heider, 2007).

The β-proteobacterium *Dechloromonas aromatica* strain RCB can degrade benzene aerobically and anaerobically with chlorate and nitrate (Coates et al., 2001). However, signature genes of anaerobic hydrocarbon activation (Heider, 2007), like the glycyl-radical enzyme benzyl-succinate synthase cluster, are missing. In contrast, the genome of strain RCB only encodes genes for the aerobic activation of aromatic compounds, including several mono- and dioxygenases (Salinero et al., 2009). Physiological experiments under nitrate-reducing conditions strongly suggest the involvement of a hydroxyl radical-mediated activation leading to phenol as primary intermediate (Chakraborty and Coates, 2005). It is quite unlikely that the very substrate-specific Cld can catalyze O_2_ production from nitrogen oxide intermediates. This possibility has been negatively tested for NO with the recombinant Cld of *Nitrospira defluvii *(Maixner et al., 2008), which was also found to be inhibited by NO (179 μM; F. Maixner and K. Ettwig, unpublished results). The open question is: Can the oxidative power for the attack on benzene come from oxygen, also under denitrifying conditions (Weelink et al., 2010)?

## OXYGEN PRODUCTION FROM NITROGEN OXIDES?

The idea that oxygen may be an intermediate of denitrifying, anaerobic bacteria emerged when the genome of the anaerobic methane-oxidizing bacterium “*Candidatus *Methylomirabilis oxyfera” was assembled from enrichment culture metagenomes.

These freshwater enrichment cultures (Raghoebarsing et al., 2006; Ettwig et al., 2009) couple complete methane oxidation with CO_2_ as the end product to the reduction of nitrite $NO_{2}^{-}$ to dinitrogen (N_2_) according to Eq. 2.

$$\begin{array}{cc} 3\,CH_{4}+8\,NO_{2}^{-}+8\,H^{+}\to 3\,CO_{2}+4\,N_{2}\,+10\,H_{2}0\left(\Delta {G^{0}}^{\prime }=-928\,kJ\,mol^{-1}\,CH_{4}\right) & \left(2\right) \end{array}$$

Methane has the second highest activation energy (after benzene) of all organic compounds. One of the prime questions was how it could be enzymatically activated under anaerobic conditions. Generally, two enzymatic activation mechanisms were already known: Aerobic methane-oxidizing bacteria (MOB) employ a monooxygenase reaction yielding methanol as the first intermediate (Hakemian and Rosenzweig, 2007; Trotsenko and Murrell, 2008). Anaerobic methanotrophic archaea (ANME), that couple methane oxidation to sulfate reduction (most likely performed in association with sulfate-reducing bacteria) reverse the last step of methanogenesis catalyzed by methyl-coenzyme M reductase (Knittel and Boetius, 2009; Scheller et al., 2010). Though energetically costly, a third possibility has also been considered – an activation mechanism involving addition of the methyl group to fumarate, catalyzed by a glycyl radical enzyme homologous to benzyl- or alkyl-succinate synthase (Thauer and Shima, 2008). 

**FIGURE 1:**
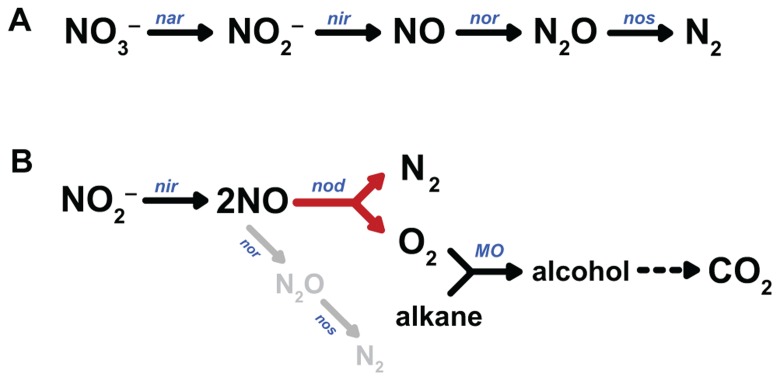
Pathways of canonical denitrification **(A)** and proposed N_2_ and O_2_ production by NO dismutation **(B)**. nar, nitrate reductase; nir, nitrite reductase; nor, nitric oxide reductase; nos, nitrous oxide reductase; nod, nitric oxide dismutase, MO, monooxygenase.

Whereas no homologues of the two last mentioned signature genes for anaerobic methane and hydrocarbon degradation could be identified in the genome, surprisingly the entire pathway of aerobic methane oxidation, starting with particulate methane monooxygenase (pMMO), was present, and prominently transcribed and expressed (Ettwig et al., 2010; Luesken et al., 2012). This was consistent with several experimental findings: The *M. oxyfera* enrichment culture was not sensitive to aerobic handling (Ettwig et al., 2009), but highly sensitive to acetylene (total inhibition at 10 μM; Ettwig et al., 2010), a known inhibitor of pMMO (Prior and Dalton, 1985). Besides methane, the *M. oxyfera* enrichment culture also oxidized other short-chain alkanes (ethane, propane, butane), a well-known activity of pMMO (Leadbetter and Foster, 1960; Hazeu and de Bruyn, 1980). Finally, using the oxidation of propylene as a proxy for pMMO activity (Prior and Dalton, 1985), comparable rates were obtained for oxygen and nitrite as electron acceptors (Ettwig et al., 2010). Also the analysis of the denitrification pathway caused surprise. In all microbial species studied so far, denitrification proceeds in a step-wise fashion, comprising the subsequent reduction of nitrate $NO_{3}^{-}$ to nitrite $NO_{2}^{-}$, nitric oxide (NO), nitrous oxide (N_2_O), and eventually dinitrogen gas (N_2_) by dedicated reductases (**Figure 1A**; Zumft, 1997; Einsle and Kroneck, 2004; Tavares et al., 2006). The last step, nitrous oxide reduction, is not always present, leaving the potent greenhouse gas N_2_O as the end product (Stein, 2011). Thus, a second startling finding was the apparent lack of an identifiable nitrous oxide reductase in the genome of *M. oxyfera*, even though it had been shown that dinitrogen gas was the end product of nitrite reduction. Despite the presence of three qNOR paralogs (see below), of which two were highly transcribed and expressed, nitrous oxide was not produced in significant amounts. Now, one of two possibilities might explain the paradoxical results: (1) activation of methane to methanol by NO, yielding N_2_ as the second product of the pMMO-catalyzed reaction, (2) the disproportionation of NO into N_2_ and O_2_ (Eq. 3), analogously to the chlorite dismutase reaction and to be catalyzed by a novel enzyme, NO dismutase (NOD). Again, such disproportionation is exergonic. 

$$\begin{array}{cc} 2NO\,\to N_{2}+O_{2}\left(\Delta G^{0^{\prime }}=-173\,kJ\,mol^{-1}O_{2}\right) & \left(3\right) \end{array}$$

Although the first possibility cannot be discarded, it is very unlikely. The pMMO of *M. oxyfera* shows a high sequence identity to the well-studied enzymes from other organisms with known crystal structures, including amino acids implicated in catalysis (Balasubramanian and Rosenzweig, 2007; Smith et al., 2011). With NO as the direct oxidizing agent, at least some modifications would be expected. Moreover, such role of NO is not in accordance with the experimental reaction stoichiometry (Eq. 2). Next, the bypass of N_2_O as an intermediate, and the formation of labeled oxygen from ^1^^8^O-labeled nitrite could be experimentally shown (Ettwig et al., 2010). The hypothetical pathway that is consistent with all observations is shown in **Figure 1B**. From the overall reaction stoichiometry (Eq. 2) it is inferred that the disproportionation of eight NO molecules would give four oxygen molecules only three of which are consumed in the activation of methane. Residual O_2_ appears to be respired by one of the terminal oxidases found in the *M. oxyfera* genome (Wu et al., 2011). Obviously, the most interesting question now is the identity of the enzyme that catalyzes oxygen and nitrogen formation from NO.

The intermediary role for oxygen in the activation of recalcitrant compounds during denitrification may not be limited to *M. oxyfera*. The facultatively denitrifying γ-proteobacterium strain HdN1 grows on a wide variety of substrates, including C6- to C20-alkanes (Ehrenreich et al., 2000; Zedelius et al., 2010). Growth on hexadecane was observed with oxygen, nitrate, or nitrite as electron acceptors, but not with N_2_O. In contrast, N_2_O did serve as a substrate for growth on the corresponding easier-to-degrade C16-alcohol and fatty acid, which do not require oxidative activation (Zedelius et al., 2010). Like *M. oxyfera*, the HdN1 genome did not contain recognizable genes for the glycyl-radical-catalyzed activation of alkanes, such as alkylsuccinate synthase. Instead, two or possibly three monooxygenases were encoded in the genome. These findings suggest that the activation of the alkane substrate in *M. oxyfera* and HdN1 take place by a similar mechanism involving oxygen, formed from nitrate or nitrite (**Figure 1B**). 

## DIVERGENT NITRIC OXIDE REDUCTASES IN *M. OXYFERA* AND OTHER DENITRIFYING MICROORGANISMS

**FIGURE 2:**
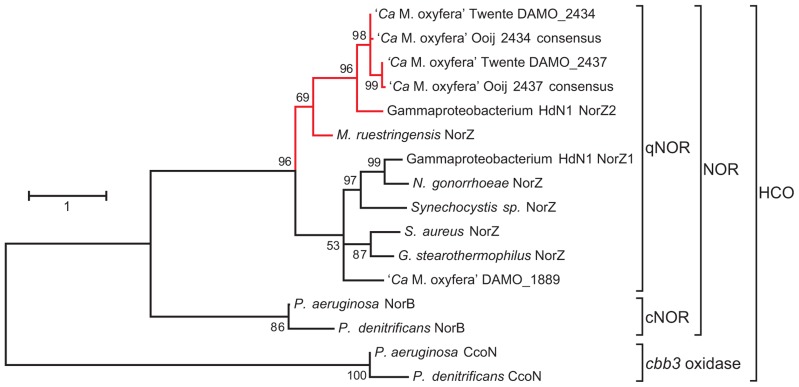
**Maximum likelihood tree (500 bootstrap replicates) of selected protein sequences from the heme-copper oxidases (HCO) superfamily, with a focus on quinol-dependent nitric oxide reductases (qNORs) including the potential NO dismutases (in red).** The tree was calculated with MEGA5 (Tamura et al., 2011) and is based on an alignment created with ClustalW using the default settings. The alignment was manually checked for correct alignment of conserved residues. The sequences Ooij 2434 and Ooij 2437 are consensus sequences based on the contigs obtained by iterative read mapping of the Ooij metagenome (SRR022748.2) to the genome of *M. oxyfera* (Dutilh et al., 2009). Accession numbers: *M. oxyfera* DAMO_2434 CBE69496, DAMO_2437 CBE69502, and DAMO_1889 CBE68939; γ-proteobacterium HdN1 NorZ1 CBL45628 and NorZ2 YP_003809511; *M. ruestringensis* G2PJH6; *N. gonorrhoeae* ZP_04723508; *Synechocystis* sp. BAA18795; *S. aureus* EGL94648; *G. stearothermophilus* 3AYF_A; *P. aeruginosa* NorB NP_249215, *P. denitrificans* NorB BAA32546, *P. aeruginosa* CcoN NP_252822; *P. denitrificans* CcoN AAC44516.

Like oxygen, NO is a strongly oxidizing compound and most microorganisms that have to deal with it as an intermediate or in their environment have developed a repertory of enzymes that convert it into the harmless N_2_O as fast as possible (Richardson, 2000; de Vries and Schröder,2002; Watmough et al., 2009). Collectively, the bacterial nitric oxide reductases (NORs) belong to the superfamily of heme-copper oxidases (HCOs; **Figure 2**). Members of the family share the presence of a heme *b* (or *a*) for electron transfer, and a second heme (*b*_3_, *a*_3_, or *o*_3_), that together with an iron (Fe_B_ in NOR) or a copper ion (Cu_B_ in oxidases) constitute the catalytic center. Both Fe_B_ and Cu_B_ are ligated to three conserved histidines. The electron-transferring heme is coordinated by two histidines as well, while one more histidine serves as the proximal ligand to the catalytic heme. This histidine sextet is a signature for HCOs. 

Nitric oxide reductases catalyze the two-electron reduction of two molecules of NO into N_2_O (Eq. 4).

$$\begin{array}{cc} 2NO\,+2H^{+}+2e^{-}\to N_{2}0\,+H_{2}0 & \left(4\right) \end{array}$$

The different NOR types are distinguished on the basis of the electron carrier that supplies nitric oxide reduction with reductant. Best characterized are cNORs which contain an additional cytochrome *c* subunit for this purpose, and qNORs which use reduced quinone (quinol) as the electron donor. Of both enzymes, atomic structures have been resolved recently (Hino et al., 2010; Matsumoto et al., 2012).

As mentioned above, the *M. oxyfera* genome contained three qNOR paralogs (EC 1.7.5.2, DAMO_1889, DAMO_2434, and DAMO_2437), in stark contrast to the lack of appreciable N_2_O production during nitrite-dependent methane oxidation (Raghoebarsing et al., 2006; Ettwig et al., 2008, 2009, 2010). DAMO_1889 was expressed in only low amounts, but the two highly similar DAMO_2434 and DAMO_2437 (84% aa identity) were among the most abundant gene products, both at the transcriptional and protein level (Ettwig et al., 2010). Detailed sequence analysis revealed that DAMO_1889 shared all important features with known qNORs, while DAMO_2434 and DAMO_2437 displayed important differences, which will be discussed in detail below. Strikingly, the unusual characteristics were consistently found in two other protein sequences available in GenBank, putative qNORs from the hexadecane-oxidizing γ-proteobacterial strain HdN1 (Zedelius et al., 2010) and from *Muricauda ruestringensis*, a Flavobacterium that had been isolated with peptone as a carbon source from a hexadecane-oxidizing, denitrifying enrichment culture (Bruns et al., 2001). A species of the same genus, *M. aquimarina*, was recently shown to degrade hexadecane and polycyclic aromatic hydrocarbons aerobically (Jiménez et al., 2011). Although the three organisms are only distantly related, their unusual qNOR-like genes form one separate cluster within the qNORs (**Figure 2**). A similar qNOR, however, is absent from the genome of the benzene-oxidizing *D. aromatica* strain RCB.

## CHARACTERISTICS OF THE PUTATIVE NO DISMUTASES

**FIGURE 3:**
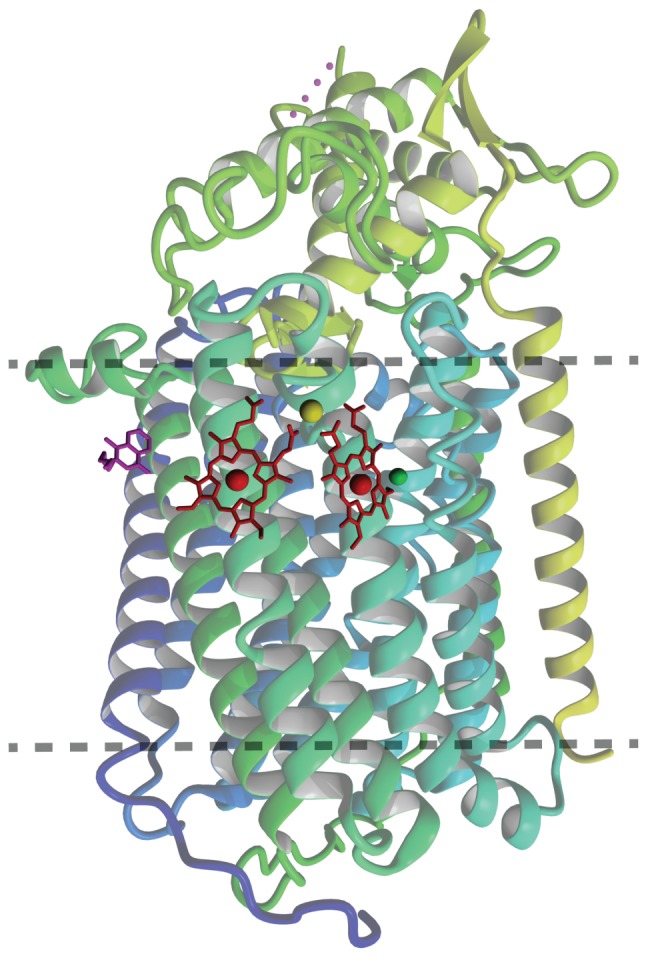
**Ribbon representation of the overall qNOR structure of *Geobacillus stearothermophilus* (3AYG, Matsumoto et al., 2012) seen from the plane of the cytoplasmic membrane (between dotted lines).** The heme groups are indicated in red, the quinol analog 2-heptyl hydroxyquinoline *N*-oxide in magenta. The zinc in the catalytic center is shown in green, and the calcium (Ca^2+^) assisting in coordination of the heme groups in pastel green.

**FIGURE 4:**
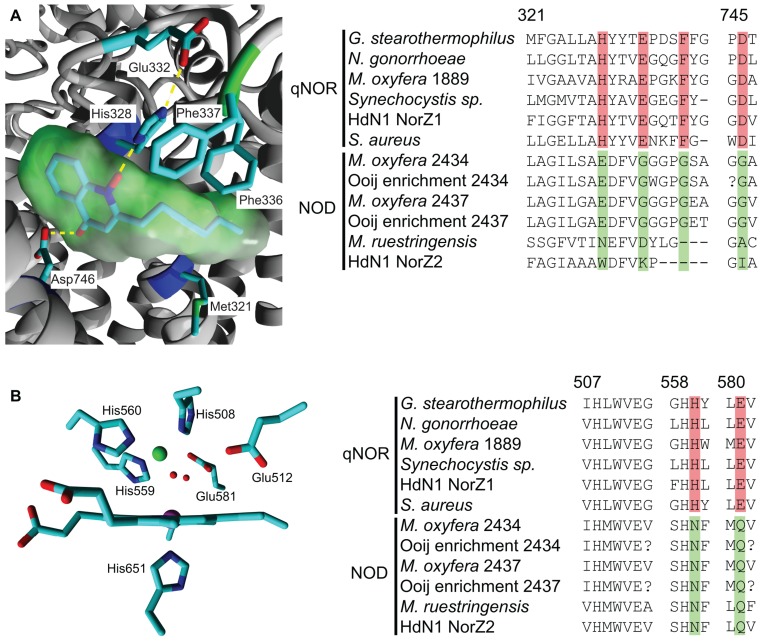
**Quinol-binding and catalytic sites in the qNOR structure of *Geobacillus stearothermophilus* (3AYG, Matsumoto et al., 2012;left) and amino acid sequence comparison of these sites in qNORs and putative NODs (right).** Sequence accession numbers and alignment are as indicated in **Figure 2**. Numbering above the alignment refers to the first amino acid and corresponds to the residue numbers of *G. stearothermophilus.* Specific changes in otherwise strongly conserved residues are highlighted. **(A)** Quinol binding site with a bound quinol analog, 2-heptyl hydroxyquinoline *N*-oxide (green molecular surface). His328 and Asp746 form hydrogen bonds with the quinol moiety and the large hydrophobic residues interact with the hydrophobic tail. **(B)** View of the catalytic site from the plane of the heme *b*_3_. The Zn_B_ is indicated in green and two water molecules in the coordination sphere of the Zn_B_ are indicated as small red spheres.

The overall atomic structure of qNOR strongly resembles the one of cNORs and other HCOs (Hino et al., 2010; Matsumoto et al., 2012). The enzyme is composed of a membrane-spanning region with 13 trans-membrane helices (TMHs) that enclose the heme *b*, heme *b*_3_, and Fe_B_ moieties, which are coordinated by the conserved histidine sextet. In the qNOR structure, the latter position is occupied by a (redox-insensitive) zinc atom, which most likely is a crystallization artifact (**Figure 3**). A particular property of qNOR is the presence of an additional (14th) N-terminal TMH that is followed by a long hydrophilic stretch of amino acids. This sequence folds at the periplasmic site as a cyt *c* domain like in cNOR, although a heme *c* itself is absent. Instead, the heme *c* position is filled by a number of voluminous aromatic amino acids. Two hydrophobic channels are observed in the structure that run parallel to the membrane and connect the hydrophobic membrane interior with the active site. These channels might function in substrate (NO) import and product (N_2_O) export. Two more features distinguish qNOR from cNOR: (1) the presence of a quinol-binding site (**Figure 4A**) and of a water-filled channel that likely plays a role in the supply of protons for NO reduction (Eq. 4; Matsumoto et al., 2012; Shiro et al., 2012). The channel leads from the bottom of the enzyme in the cytoplasm up to the catalytic site.

The sequence comparison of the *M. oxyfera* and the other unusual qNORs establish both resemblances and significant differences with respect to canonical qNORs. In DAMO_1889, all characteristics are conserved, suggesting the protein to be a genuine qNOR. Also in DAMO_2434, DAMO_2437, and their relatives the overall folding is apparently maintained with respect to the one of qNORs, as is inferred from sequence comparison and structural modeling using qNOR of *Geobacillus stearothermophilus* (PDB 3AYF and 3AYG) as the template (not shown). The arrangement of the 14 TMHs, the hydrophilic domain devoid of heme *c*, all histidines except one, both putative substrate channels and a portion of the amino acids related with the H^+^ channel are conserved. This suggests that DAMO_2434 and its relatives, hereafter referred to as putative NOD, bind the electron-transferring heme *b*, the catalytic heme *b*_3_, and non-heme iron (or another catalytic metal). However, in the NODs one of the coordinating histidines is consistently replaced by an asparagine (**Figure 4B**). Similarly, a glutamate in close vicinity to the catalytic center, which has been implied with catalysis (Thorndycroft et al., 2007; Flock et al., 2009; Hino et al., 2012; Shiro et al., 2012) is substituted by a glutamine residue. Also the amino acids lining the proposed H^+^ channel in qNOR have undergone several substitutions in the putative NODs. Most importantly, the unusual “qNORs” lack a proper quinol-binding site. Conserved residues that are assumed to constitute the quinol-binding site in qNORs are substituted for amino acids that are unlikely to provide a suitable site for quinol binding in the putative NODs (**Figure 4A**). In summary, the latter apparently are unable of accepting external electrons, they have a different catalytic site and might be impeded in H^+^ uptake from outside the protein. Obviously, these properties compromise a role as nitric oxide reductases. The question then is what they do, presuming that they do bear an important biological function – a reasonable assumption given their high expression levels in *M. oxyfera*. It is tempting to speculate that the modified proteins can bind two NO molecules, rearrange N-O bonds with the aid of the hemes and non-heme metal (iron or otherwise), and recombine both N and O atoms such that N_2_ and O_2_ are made. In other words, the enzymes would act as an NO dismutase. At this stage, this is speculation. The proof can only come from the purification and rigorous characterization of these intriguing enzymes.

## Conflict of Interest Statement

The authors declare that the research was conducted in the absence of any commercial or financial relationships that could be construed as a potential conflict of interest.
